# Effect of low versus high frequency stimulation on freezing of gait and other axial symptoms in Parkinson patients with bilateral STN DBS: a mini-review

**DOI:** 10.1186/s40035-017-0083-7

**Published:** 2017-05-17

**Authors:** Tao Xie, Mahesh Padmanaban, Lisa Bloom, Ellen MacCracken, Breanna Bertacchi, Abraham Dachman, Peter Warnke

**Affiliations:** 1 Department of Neurology, University of Chicago Medicine, Chicago, IL USA; 2 Speech and Swallowing Section, Department of Surgery, University of Chicago Medicine, Chicago, IL USA; 3 Department of Radiology, University of Chicago Medicine, Chicago, IL USA; 4 Department of Neurosurgery, University of Chicago Medicine, Chicago, IL USA

**Keywords:** Low frequency stimulation, Freezing of gait, Swallowing, Speech, Axial symptoms, DBS, STN, Parkinson’s disease

## Abstract

Some studies have shown that low frequency stimulation (LFS, most commonly 60 Hz), compared to high frequency stimulation (HFS, most commonly 130 Hz), has beneficial effects, short-term or even long-term, on improving freezing of gait (FOG) and other axial symptoms, including speech and swallowing function, in Parkinson disease (PD) patients with bilateral subthalamic nucleus deep brain stimulation (STN DBS). However, other studies failed to confirm this. It seems not clear what determines the difference in response to LFS. Differences in study design, such as presence or absence of FOG, exact LFS used (60 Hz versus 80 Hz), study size, open label versus randomized double blind assessment, retrospective versus prospective evaluation, medication On or Off state, total electric energy delivered maintained or not with the change in frequency, and the location of active contacts could all potentially affect the results. This mini-review goes over the literature with the aforementioned factors in mind, focusing on the effect of LFS versus HFS on FOG and other axial symptoms in PD with bilateral STN DBS, in an effort to extract the essential data to guide our clinical management of axial symptoms and explore the potential underlying mechanisms as well. Overall, LFS of 60 Hz seems to be consistently effective in patients with FOG at the usual HFS in regards to improving FOG, speech, swallowing function and other axial symptoms, though LFS could reduce tremor control in some patients. Whether LFS simply addresses the axial symptoms in the context of HFS or has other beneficial effects requires further studies, along with the mechanism.

## Background

Freezing of gait (FOG) is a disabling clinical phenomenon characterized by brief episodes of inability to step or by extremely short steps that typically occur on initiating gait or on turning while walking, with variability in gait metrics between FOG episodes and a substantial reduction in step length with frequent trembling of the legs during episodes [1]. Physiological studies, functional imaging, and clinical–pathological studies localize the disfunction in frontal cortical regions, basal ganglia, and midbrain locomotor region as the probable origins of FOG [1]. FOG occurs more at dopaminergic medication Off than On state, though some FOG even becomes worse with more dopaminergic medications [1]. Deep brain stimulation (DBS) of the subthalamic nucleus (STN) at usual high frequency stimulation (HFS) can alleviate some FOG in some patients, particularly that related to medication Off state, while DBS can also create FOG in others with Parkinson’s disease (PD) [2, 3].

Low frequency stimulation (LFS, most commonly 60 Hz), compared to HFS (most commonly 130 Hz), has been shown to have beneficial effects on improving FOG and other axial symptoms, such as speech and swallowing function, in PD patients with bilateral STN DBS in some studies [4–10], or selected patients [11], but not in others [12–15]. Some found short-term but not long-term beneficial effect [16], while others found both short-term and long-term benefits after 6 weeks, 8 months and even 10 months study periods [4, 6, 7]. It is not clear what determines the difference in the response of FOG and other axial symptoms to LFS. Differences in study design, such as the presence or absence of FOG in evaluation or history, exact LFS used (60 Hz versus 80 Hz), study size, open label versus randomized double blind assessment, retrospective versus prospective evaluation, medication On or Off state, status of total electric energy delivered (TEED) maintenance with frequency adjustment [17], and the location of the active contacts (ventral versus dorsal) used could all potentially affect the results or its interpretation.

This mini-review tries to go over these studies in comprehensive detail with the aforementioned factors in mind, focusing on the effect of LFS versus HFS on FOG and other axial symptoms in PD with bilateral STN DBS, and evaluate and summarize the current literature to guide our clinical practice in managing axial symptoms and also explore the underlying mechanism within the limitations of the studies. References for this review were identified through PubMed searches with the combination of the search terms “frequency (or low-frequency or high-frequency)”, “DBS (or STN, STN-DBS, subthalamic or deep brain stimulation)”, “freezing (or gait, balance, axial or just leaving blank to expand the searching)”, and “PD (or Parkinson’s disease or Parkinson disease)”. We also identified articles through the authors’ references of pertinent articles. Only articles in English were reviewed. The choice of articles to include in this review was based on the quality of each study and the pertinence to the topics reviewed here.

## LFS (60 Hz) versus HFS (130 Hz) in patients with FOG

Moreau et al. first studied 13 Parkinson patients, who developed axial symptoms (gait hypokinesia and postural instability, all having FOG) after a mean of 5 years on bilateral STN DBS [4]. They evaluated the Stand-Walk-Sit (SWS) test and Unified Parkinson Disease Rating Scale section III for motor function (UPDRS-III) at medication Off state in a randomized, double blind, prospective cross over study, under the condition of 1) DBS Off, 2) HFS (130 Hz) with usual voltage (median 3.0 V), 3) HFS (130 Hz) with higher voltage (median 3.7 V), 4) LFS (60 Hz) at equivalent energy with usual voltage as in condition 2, and 5) LFS (60 Hz) at equivalent energy with higher voltage as in condition 3. Monopolar stimulation was performed with the contact that had given the best clinical results over the previous years: ventral contacts (contact 0 or 1) were used in 10 patients, and dorsal contacts (contact 2 or 3) were used in 3 patients. Conditions 4 and 5 were found to be significantly better than conditions 1, 2, and 3 in all aspects of SWS test, including time and number of steps, and the number of FOG spells before completion, with best gait function and least FOG obtained at setting 5 for all the parameters. In condition 3, all parameters worsened as compared to condition 2﻿, with more FOG spells when compared to DBS Off state. No significant difference was found between130Hz with usual voltage as in condition 2 and 60 Hz at equivalent energy with higher voltage as in condition 5 in UPDRS-III scores, and both were significantly better than DBS Off. After 8 months, the clinical benefit of condition 5 on gait was still maintained in 85% of patients though they did require slightly higher daily dose of levodopa to achieve the same clinical benefit for segmental parkinsonian symptoms. Two patients had to switch back to 130 Hz due to a worsening of segmental symptoms, especially the tremor. Overall they found a negative effect on FOG with increasing voltages (median 3.7 V) at HFS of 130 Hz and a beneficial effect of LFS of 60 Hz at equivalent delivered energy levels, with the greatest benefit on FOG in the 60 Hz high- voltage equivalent-energy condition.

Brozova et al. studied 12 PD patients status post bilateral STN DBS who had persistent gait difficulties (7 with FOG, 6 with postural instability, and 7 with falls) after a mean of 4.3 years on bilateral HFS [11]. Three patients could not tolerate LFS due to worsening of tremor (one), rigidity (one) and gait (one). They checked UPDRS scores after 8–12 weeks on the rest of the 9 patients in an open label study after switching to 60 Hz and compared to HFS at medication On state. Overall significant improvements were observed in the UPDRS-II subscale and individual items of speech, falling and walking, and UPDRS-III items for speech, and gait, which were all considered axial symptoms. Individually, postural instability and gait were found worsened in two patients, and an average increase of 1.3 V was required bilaterally in 7 patients for maintenance of beneficial effects in other PD symptoms. The different responses made them conclude that LFS might not be the solution for all gait problems in advanced PD. It was not mentioned whether or not TEED was maintained, or whether patients with FOG had a better response to the LFS treatment than others.

Since all of these patients cited developed FOG and other axial symptoms on average 4–5 years after bilateral STN DBS [4, 11], the question arose as to whether chronic HFS or disease progression was responsible for the development of FOG. Xie et al. reported two PD patients who developed FOG upon activation of the newly placed STN DBS at routine HFS [6]. FOG and UPDRS-III scores at LFS of 60 Hz and HFS of 130 Hz at both medication Off and medication On states were assessed. Both patients showed immediate improvement in UPDRS-III score, FOG and speech at LFS regardless of medication Off and On state. The beneficial effects lasted for 10 months of the study period. This study not only demonstrated an immediate effect of DBS frequency on FOG, factoring out previously confounding variables of chronic stimulation effect, disease progression or a combination of the two, but also demonstrated improvement in other axial symptoms, such as speech, under LFS in the studied subjects.

Similarly, Ramdhani et al. retrospectively studied 5 patients, all of whom had Off medication FOG and 3 of whom also had concurrent medication refractory FOG before the surgery [10]. Their FOG and axial symptoms worsened on HFS (130-185 Hz) of bilateral STN so they were subsequently switched to LFS (60 Hz) within an average of only 2 months after their DBS surgery. UPDRS-III along with subscores for axial and gait symptoms and FOG were assessed. Under HFS (130-185 Hz), 4/5 patients developed FOG at medication On state, while under LFS only 2/5 patients had FOG, with reduced severity and axial symptoms, along with amelioration of their segmental symptoms and levodopa induced dyskinesia. Consistent with the report by Xie et al. [6], these series also demonstrated the success of LFS compared to HFS on FOG shortly after the bilateral STN DBS surgery. All patients had activation of ventral contacts with 3 requiring simultaneous dorsal contacts stimulation. It was not mentioned whether or not TEED was adjusted with change in frequency in these patients.

Xie et al. further studied 7 PD patients with bilateral STN DBS of 4.4 years duration on average and medication refractory FOG at 130 Hz stimulation in a randomized, double blind, prospective crossover manner at medication On status [7]. SWS test (number of FOG spells and time to finish the 7-m round trip timed walking test) and FOG questionnaire were assessed for both objective and subjective measurement of FOG and UPDRS-III was utilized for other axial and motor symptoms. HFS (130 Hz), LFS (60 Hz) and DBS Off conditions were randomly assigned, with usual voltage, pulse width and contacts without adjusting the TEED. Condition producing the least FOG (60 Hz in all patients) was then left on for 6 to 8 weeks to repeat all the tests at 60 Hz stimulation and medication On state. They found that LFS of 60 Hz stimulation improved FOG, axial symptoms and overall parkinsonism compared with 130 Hz. Both the axial score and UPDRS-III total motor score were also significantly better at LFS compared with the DBS Off state. The axial score was worse at HFS compared with the DBS Off state, as similarly seen in other studies at medication On state [18–20]. There was no statistically significant difference in any of the measurements under LFS between the initial visit and the follow-up visit 6 weeks later on average, suggesting that the benefits obtained at LFS persisted over the 6-week period studied. One patient had to be switched back to 130 Hz 3 weeks later due to the worsening of his hand tremor.

## LFS (all or mostly 80 Hz) versus HFS (130 Hz) in patients with general gait impairment or axial symptoms

LFS was also explored in other studies for a long-term effect but mainly with 80 Hz stimulation. Ricchi et al. studied 11 PD patients who developed gait impairment (frequent falls and/or freezing of gait) within 5 years after DBS surgery [16]. SWS, UPDRS-II, −III, and clinical global improvement scale were assessed. Patients were assessed at baseline and 3 h after switching the stimulation frequency to LFS of 80 Hz. Follow-up evaluations were carried out after 1, 5, and 15 months on 80 Hz at medications On state. Equivalent TEED was maintained. Significant improvement on SWS test in the number of steps after acutely switching to 80 Hz was found, with no deterioration of PD segmental symptoms. However, gait improvement was no longer detectable by the SWS test at follow-up evaluations 1, 5, and 15 months later. Three patients were switched back to 130 Hz because of unsatisfactory control of motor symptoms (including worsening tremor in two of them). Eight patients were maintained at 80 Hz for up to 15 months, with five showing a global improvement and three no change. They concluded that the 80 Hz stimulation may have immediate effect on gait improvement, but it may not be maintained over a long period of time. It is worth noting that there was no FOG elicited with either the 80 Hz or 130 Hz stimulation in this study, even though some patients had a history of the FOG. Monopolar stimulation of the dorsal contacts was performed in all but one patient for whom ventral contacts were used.

Sidiropoulos et al. studied 45 PD patients with DBS [13], all but one having bilateral STN stimulation, with one of them also having a third electrode placed in the left globus pallidus interna, two having unilateral pedunculopontine nucleus (PPN) stimulation, one having undergone fetal mesencephalic tissue grafting in the past, and one having a previous right pallidotomy. They all met one of the following criteria: (a) early loss of axial improvement (<1 year) after STN DBS; (b) loss of axial benefit after several years of STN DBS; (c) no satisfactory benefit from conventional HFS despite optimization of medical treatment and physiotherapy; (d) severe hypophonia, alone or in conjunction with other axial symptoms. Primary outcome was UPDRS-III and axial and gait subscores; secondary outcomes were speech subscores and self-reported number of falls. They were assessed at median of 111.5 days after the switch, at medication On state without TEED maintained, with HFS (130-185 Hz) and LFS (39 switched to 80 Hz and 6 to 60 Hz). They found no significant difference in any of the outcomes measured, with some benefit in some patients subjectively, but not evident objectively. Worth noting, FOG was not a stated prerequisite in the enrollment of patients.

## LFS (60 Hz) versus HFS (130 Hz) in patients, mostly with FOG in history

Khoo et al. studied 14 PD patients with bilateral STN DBS, with primary outcome being difference in HFS and LFS in UPDRS-III score, and secondary outcome in UPDRS-III akinesia and axial motor subscores, gait by the 10-meter timed walk test at preferred pace, and the postural stability by the Berg Balance Scale [9]. They first verified optimal contacts at HFS (130 Hz) and LFS (60 Hz) defined by the best UPDRS scores in monopolar settings with each frequency. This was followed by a further evaluation at HFS and LFS with optimal contacts used on each setting, at medication On state, in a randomized, double blind, prospective cross over study, with TEED maintained as later clarified [21]. Optimal contact positions were significantly different for HFS and LFS, with LFS being more ventrally distributed. Mean UPDRS III score was less (meaning better motor function) for LFS than HFS with optimal contacts. Immediate effect of the LFS (1 h after each condition) demonstrated significantly less severe axial motor signs and akinesia scores, less time and fewer steps to complete the walk, and a tendency of improving the balance, compared to HFS. No significant difference between HFS and LFS was observed in tremor or rigidity in their study. Interestingly, none of them had FOG on assessment at medication On state, though most of them had FOG at medication Off state by history.

## LFS (60 Hz or below) versus HFS (≥130 Hz or >100 Hz) in patients without predefined FOG state

The effect of LFS vs HFS was also assessed on general PD patients without pre-defined FOG state. Stegemoller et al. studied 17 PD patients with bilateral STN DBS at least 6 months after the surgery, who were classified as tremor dominant (8 patients) or non-tremor dominant patients (9 patients) [14]. UPDRS-III, gait (8-meter walkway), balance (force plate), and verbal fluency were measured. DBS Off state, LFS (60 Hz), and HFS (≥130 Hz) were randomly assigned and compared at medication Off state. Normal voltage and pulse width were maintained hence the TEED was not maintained with the frequency change. They concluded that HFS significantly reduced tremor in tremor dominant patients, but there were no acute differences (as assessed 10 min after each test condition) between LFS and HFS on gait, balance, and verbal fluency in both tremor and non-tremor groups. The FOG state in these patients was not mentioned.

Vallabhajosula et al. studied 19 PD patients with bilateral STN DBS without pre-defined FOG state [15]. UPDRS III scores, static and dynamic postural control using gait initiation and gait evaluations were assessed in three conditions at medication Off state: DBS Off, LFS (60 Hz), and HFS (>100 Hz). The baseline voltage was stable across conditions, hence TEED was not maintained with the change in frequency. Additionally 10/19 participants were also stimulated at 30 Hz and 60 Hz and at higher voltages. They found that the UPDRS-III score, step length and velocity during gait initiation and gait speed significantly improved during 60 Hz and >100 Hz conditions when compared to the Off condition. There were no significant differences between 60 Hz and >100 Hz conditions, and using LFS at higher voltage showed no improvement over >100 Hz condition. They concluded that the positive effects of both LFS and HFS on postural control and gait were similar and clinical changes were relatively small and that LFS may not help improve postural control and gait particularly for persons with PD who do not develop gait-related disorders after HFS. Again, the FOG state in these patients was unknown, and presumably not many patients with FOG were in their study group by design.

## Effect of TEED on the effect of LFS versus HFS

TEED was a commonly debated issue in several publications [8, 22–25]. Some studies had TEED maintained [4, 5, 8, 9, 16], but others did not [6, 7, 12–15]. Phibbs et al. studied 20 PD patients who were at least 3 months after on bilateral STN DBS with changes in gait (7 with balance issues, 7 with FOG, 6 with festination) [12]. SWS, UPDRS III, and GaitRite (gait evaluation) were checked. The primary outcome was the change in stride length as measured by GaitRite. Secondary outcomes included the time on the SWS test, other gait parameters collected on the GaitRite including velocity, cadence, single and double limb support time, and the ratio of the single and double limb support time. HFS (130 Hz) and LFS (60 Hz) were assessed, at medication Off state. TEED was not maintained with change in frequency. No significant difference was found in primary outcome of stride length with the change in frequency or the secondary measures. This study did not demonstrate improved gait with lower frequency stimulation, but this might have been due to decreased energy delivered from the lower frequency without TEED maintained, according to the authors of the study. Worth noting, the gait complaints were found hard to be replicated during the study, as stated in the paper. Nevertheless, the patient with FOG at 130 Hz still had a better gait at 60 Hz. Xie et al. reported beneficial effect of LFS on FOG and swallowing function in patients with medication refractory FOG at HFS [7]. Some wondered whether the beneficial effect of the LFS was due to the reduced energy as TEED was not adjusted [23, 25]. In a follow up study, they evaluated the effect of LFS on FOG with and without adjusting the TEED in a double-blind design and reached the same conclusion on the beneficial effect of LFS on FOG and axial symptoms regardless of the TEED adjustment [8], suggesting that it is the LFS that brought the benefit, not the reduced energy, in that study.

## LFS versus HFS on speech

LFS may also have beneficial effect on speech. Moreau et al. studied 11 PD patients, who were on an average of 5 years status post bilateral STN DBS, and selected on the basis of gait disorders (FOG and/or gait hypokinesia) [5]. Speech intelligibility and aerodynamic and acoustic parameters were measured in a double blind, randomized conditions of DBS settings of Off, 60 Hz and 130 Hz stimulation, at medication Off state with TEED maintained. The authors found an improvement in aerodynamic speech parameters during LFS of 60 Hz accompanied by significant clinical benefit of more intelligible speech compared to the HFS and Off DBS state. Xie et al. also similarly found improved speech at LFS in patients with FOG at HFS [6].

## LFS versus HFS on swallowing function

LFS also has beneficial effect on swallowing function. Xie et al. first studied 7 PD patients with bilateral STN DBS of 4.4 years duration on average and medication refractory FOG at 130 Hz frequency in a randomized, double blind, prospective crossover study at medication On state [7]. The Penetration-Aspiration scale was used to assess objective swallowing function in videofluoroscopic swallow test and a swallowing questionnaire was used to assess subjective swallowing function after the swallow test. SWS test (number of FOG spells and time to finish the test) and FOG questionnaire for both objective and subjective measurements on FOG, and UPDRS-III for axial and other motor symptoms were also assessed. HFS (130 Hz), LFS (60 Hz) and DBS Off conditions were randomly assigned, with usual voltage, pulse width and contacts hence TEED was not maintained. The condition producing the least FOG (60 Hz in all patients) was then left on for an average of 6 weeks to repeat the videofluoroscopic swallow test, swallowing questionnaire, and other clinical assessment under 60 Hz stimulation and medication On state. The study found that LFS significantly reduced the aspiration frequency on objective videofluoroscopic swallow test by 57% and significantly reduced subjective swallowing difficulty on questionnaire by 80% compared to HFS. The beneficial effect of the LFS remained persistent over the 6-week period studied. This study also showed that 60 Hz stimulation improved FOG, axial symptom and overall parkinsonism compared to 130 Hz.

The key articles reviewed above are summarized in Table 1.

**Table 1:** Summary of publications evaluating the effects of LFS of STN on FOG and other axial symptoms

Ref#	Author, year	Number of patients	Study design	Med On/Off	FOG at HFS	LFS/HFS value	Follow up duration	TEED adjustment	Outcomes
[4]	Moreau et al., 2008	13	Randomized double blinded	Off	Yes	60 Hz/130 Hz	8 months	Yes	Acutely, significantly better in all aspects of SWS, including FOG at LFS. F/U: 85% pts. had maintenance of clinical benefit on gait 8 months after on LFS. 10 pts. on ventral, 3 pts. on dorsal contacts. 2 pts. switched back to HFS due to worsening tremor.
[11]	Brozova et al., 2009	12	Non-randomized non-blinded	On	Yes (7/12 pts)	60 Hz/HFS (unknown exact Hz for HFS)	8–12 weeks in 9 pts. (3 pts. unable to stay on LFS due to worsening of tremor, gait, and rigidity)	Not mentioned but voltage increased in 7 pts	F/U: overall, improvements in speech, falling and walking in UPDRS-II and speech and gait in UPDRS-III subscores but worsening of postural stability and gait in 2 pts.
[6]	Xie et al., 2012	2	Non-randomized non-blinded	Both	Yes	60 Hz/130 Hz	10 months	No	Acutely: improvements in UPDRS-III score, FOG and speech at LFS at medication Off and On state. F/U: the beneficial effects lasted for 10 months of the study period.
[10]	Ramdhani et al., 2015	5	Non-randomized non-blinded	On	Yes (4/5)	60 Hz/130–185 Hz	2–6 months	Not mentioned	F/U: at HFS 4/5 had FOG while at LFS only 2/5 pts. had FOG, with reduced severity and axial symptoms, along with amelioration of segmental symptoms and levodopa induced dyskinesia. All pts. had ventral contacts and 3 pts. had simultaneous dorsal contacts.
[7]	Xie et al., 2015	7	Randomized double blinded	On	Yes	60 Hz/130 Hz	6 weeks	No	Acutely: compared with HFS, LFS improved swallowing function, FOG, and axial and overall parkinsonism. The axial score and UPDRS-III score also improved at LFS compared to DBS Off. The axial score was worse at HFS. F/U: benefits persisted over the 6-week study period. 1 pt. switched back to 130 Hz due to worsening tremor.
[16]	Ricchi et al., 2012	11	Non-randomized blinded and non-blinded portions	On	No FOG on exam, some had FOG in history	80 Hz/130 Hz	1, 5 and 15 months	Yes	Acutely: improvement on SWS test after acutely switching to LFS, with no deterioration of segmental symptoms. F/U: gait improvement no longer detectable by the SWS test 1, 5, and 15 months later. 3 pts. switched back to HFS because of unsatisfactory control of motor symptoms (tremor in 2). 8 pts. maintained at LFS for up to 15 months, with 5 showing a clinical global improvement on the scale. All on dorsal except 1 on ventral contacts.
[13]	Sidiropoulos et al., 2013	45	Non-randomized non-blinded	On	Not specified, but axial impairment with no satisfactory benefit from HFS	60–80 Hz/130–185 Hz (6 on 60 Hz, 39 on 80 Hz)	111.5 days, (the median, up to 4 years)	No	F/U: overall, no improvement with LFS on any of the measures in UPDRS III motor, axial, gait, and speech subscores, and self-reported number of falls.
[9]	Khoo et al., 2014	14	Randomized double blinded	On	No FOG at medication ON assessment, but most had FOG at medication OFF by history.	60 Hz/130 Hz	No long term follow up	Yes	Acutely: mean UPDRS III score, axial motor subscore and akinesia subscores were improved for LFS. Also, less time and fewer steps to complete the 10-m walk and a tendency of improving the balance. No difference between HFS and LFS in tremor or rigidity. Optimal contacts for LFS were more ventrally distributed.
[14]	Stegemoller et al., 2013	17	Randomized double blinded	Off	Not specified	60 Hz/≥130 Hz	No long term follow up	No	Acutely: HFS significantly reduced tremor in tremor dominant (TD) pts., but no acute differences between LFS and HFS on gait, balance, and verbal fluency in both TD and non-TD pts.
[15]	Vallabhajosula et al., 2015	19	Randomized, blinded and non-blinded portions	Off	Not specified	Phase 1: Off stim, optimal with HFS (≥100 Hz), LFS (60 Hz) without TEED maintained Phase 2 (10/19 pts): Off stim, very LFS (30 Hz) with highest tolerable voltage, LFS (60 Hz) with highest tolerable voltage, HFS at baseline voltage	No long term follow up	No	Acutely: UPDRS-III score, step length and velocity during gait initiation and gait speed improved during LFS and HFS when compared to the DBS Off condition. No significant differences between LFS and HFS conditions. Using LFS at higher voltages showed no improvement over HFS condition.
[12]	Phibbs et al. 2014	20	Randomized double blinded	Off	Yes (7/19 pts) per history but hard to replicate on exam	60 Hz/130 Hz	No long term follow up	No	Acutely: no significant difference was found in primary outcome of stride length with the change in frequency or the secondary measures (time on SWS test, data on gait from GaitRite). Improved FOG at LFS in the pt. with FOG at HFS, per description.
[5]	Moreau et al., 2011	11	Randomized double blinded	Off	Yes	60 Hz/130 Hz	No long term follow up	Yes	Acutely: improvement in aerodynamic speech parameters during LFS accompanied by significant clinical benefit of more intelligible speech compared to HFS and DBS Off states

Notes: *LFS* low frequency stimulation, *HFS* high frequency stimulation, *FOG* freezing of gait, *Med* medications, *TEED* total electrical energy delivered, *SWS* stand walk sit test, *Pts* patients, *Ref*# reference number, *F/U* follow up.

## Discussions

One of the important questions is how we can determine which patients are potential responders to LFS. FOG at HFS seems to have the most consistent response to LFS so far, as almost all of the studies with patients exhibiting FOG at HFS on exam during the study showed a good response to LFS [4–8, 10], regardless of medication Off [4–6] or On state [6, 7, 10], and with [4, 5, 8] or without adjusting the TEED [6–8]. Some with FOG in history (medication Off) but not necessarily on exam (medication On) during the study also had good or some response to LFS [9, 16]. There was an overall good response to LFS in a study that only some patients had FOG [11]. There was also a case of FOG at 130 Hz who responded well to 60 Hz even though the overall response of that study to LFS was negative [12]. Studies that did not specify FOG as a pre-requisite for the enrollment or in which presumably the majority of the patients did not have FOG during the exam or in their history usually had no significant beneficial effect under LFS compared to HFS [12–15]. This is possibly because there is no significant benefit of LFS in these without FOG or the effect of LFS on a small number of participants with FOG is hard to reach statistical significance. It is also consistent with the thought that LFS probably would not be of benefit if there is no gait problem at HFS [15].

The other axial symptoms in patients with FOG at HFS, such as speech [5, 6], swallowing [7], and the axial symptoms measured in UPDRS-III [4, 7, 9], also respond well to LFS. Whether one adjusted for TEED or not does not seem to affect the beneficial response of LFS in FOG [8], particularly with a normal range of DBS parameters [7], as frequency is thought to be more important than the total energy delivered [22]. How can this paradoxical result of the TEED be explained? We think, in one way, the reduction in energy could possibly lead to less non-specific stimulation or less overstimulation to the adjacent structures of the brain, which might lessen the FOG (if FOG is due to overstimulation), meaning that maintenance of the TEED would make LFS less effective for relief of the FOG. On the other hand, there has been a study showing that LFS was more beneficial for FOG when used in conjunction with higher voltage [4], meaning that the maintenance of the TEED could make LFS more effective for relief of the FOG. The two-way change probably explains why there is no difference with and without TEED adjustment, as tested in both conditions in the same group of patients [8]. Certainly with the ability to now perform directional DBS with the new lead configurations available, this problem can be further deciphered, specifically whether stimulation of surrounding structures - and if so which – contributes to FOG and to what extent.

Hence, the presence of FOG at HFS seems to be a good indicator to determine whether the patient will have a good response of FOG, speech and swallowing function and other axial symptoms to LFS [4–8, 10].

The exact frequency of the LFS used, most commonly defined as 60 Hz or alternatively the low-to-intermediate 80 Hz, might also possibly make a difference in outcomes, with all successful responses of FOG to LFS stemming from 60 Hz stimulation [4–8, 10], but lack of response [13], or lack of persistent response from 80 Hz (or mainly 80 Hz) stimulation [16], though other reasons as discussed also could contribute to this disparity. Successful responses to 60 Hz stimulation makes sense if the stimulation of the PPN is considered as part of the mechanisms as to be discussed in further details below, given the report that stimulation of the PPN’s descending projections using 60 Hz causes a “push” toward locomotion [26], while 80 Hz was arguably reported to produce negative effect on major motor symptoms [27].

The position of the active contact might also make difference. Ventral contacts were more commonly found in those with benefit from LFS according to some studies [4, 9, 10], but not in others [6, 7, 16]. Some found anteriorly or dorsal-anteriorly misplaced STN electrode as a cause [28, 29].

We don’t know how long the benefit from LFS on axial symptoms can last. There is evidence of long lasting benefit, ranging from 6 weeks to 10 months of studied periods being reported [4, 6, 7]. Short-term but not long-term benefit was also reported in one study, though the majority of the patients used 80 Hz as LFS, with no FOG being elicited in either 80 Hz or 130 Hz stimulation during the evaluation but a history of the FOG [16].

Another important caveat to bear in mind when interpreting the data is the medication status. For example, Xie et al. examined patients in the medication On state [7], so the readers should not expect to see a significant improvement of the total motor score with HFS compared to DBS Off [30], because it is widely acknowledged that the improvement of all motor symptoms by DBS (at least at commonly used HFS) was very close, or equal, to the best levodopa response [31]. This point is also consistent with many other classic or large data studies [18–20]. Similarly, there would not be much change in tremor scores in medication ON state across different stimulation settings as the tremor is levodopa responsive in most of their cases. The study by Khoo et al. reaffirmed this point as there was no difference in tremor score between LFS and HFS at medication On state [9].

We need to clarify that you don’t need LFS if there is no FOG at HFS, as STN DBS significantly improves FOG at the medication Off and stimulation On condition (mostly at routine HFS) compared with baseline at medication Off in meta-analysis [32], although in a prospective study, FOG at baseline was still present in 45% of PD patients treated with HFS of the STN at 6 and 12 months [3]. Also HFS is more effective in controlling tremor and other segmental symptoms in general [33].

Most FOG seems to occur under chronic stimulation 4–5 years after bilateral STN DBS placement [4, 5, 7, 11, 16], which is consistent with the worsening of the axial symptoms as time goes on [18–20]. Is it due to disease progression or chronic stimulation or both? They are all possible. One of the possibilities is the deterioration of the non-dopaminergic system as the disease progresses, particularly around 15 years after disease onset [34], which is equivalent to 4–5 years (or even 2 years) after the STN DBS, given the average time of DBS placement is about 11–13 years after PD onset [35–37]. The non-dopaminergic cholinergic system has been found to be associated with gait dysfunction [38, 39]. Another possibility is that the chronic stimulation is associated with gradual escalation in the voltage over time, which eventually could produce relatively large volume stimulation strong enough to affect axial symptoms, as the axial symptoms seem to be specifically sensitive to HFS [4, 8, 9], and high energy could be more likely to cause FOG [4]. However, this is not necessarily always the case, as FOG could also occur under acute stimulation settings [6], or a few months after the STN DBS placement [10], even at normal range of parameters and seemingly properly placed STN DBS electrodes (at least under current understanding) [6]. It is most likely a frequency specific response of axial symptoms to HFS [4, 8, 9], as HFS has been consistently shown to be beneficial for the segmental symptoms of Parkinson’s [33], particularly for tremor [4, 7, 11, 14, 16]. Does the LFS only resolve the axial symptoms in the context of HFS? It might be a bit premature to determine, as there is a significant beneficial effect of LFS on axial and total motor scores when compared with the DBS Off state, in addition to the beneficial effect of LFS compared to HFS on swallowing function and FOG as reported [7], although the statistical power is limited by the small number of subjects in the study. Similar beneficial effects of LFS compared with DBS Off state were also reported by other studies in patients with FOG [4, 5]. Overall, the number of studies available on the effect of LFS versus DBS Off state in patients with FOG or other axial symptoms is still limited, which probably makes it hard at present to assess the exact effect of LFS beyond the comparison with HFS.

Currently, there are two major theories for the underlying mechanism. One is the spread of the current to the PPN area (PPNa), which is close to STN (only about 5–8 mm away) with physiological and anatomical connections [40]. Functional imaging also points to the PPNa as the functional control region of FOG [41]. HFS of STN DBS could exert unfavorable effects on these areas. Extreme LFS of 10 Hz to 25 Hz stimulation of the human PPNa improves gait [27, 42, 43], whereas 80 Hz was arguably reported to produce negative effect on the major motor symptoms. Stimulation of the PPNa’s descending projections using 60 Hz has been shown to cause a “push” toward locomotion [26]. These suggest that 60 Hz probably would be more likely to produce a beneficial effect in FOG than 80 Hz or higher frequencies. Another possible mechanism is that LHS of 60 Hz might override the abnormal neuronal oscillation and boost the prokinetic gamma band activity [44, 45]. Speech and swallowing functional areas are also thought to be in the area surrounding PPNa [46], which is probably why they share a similar beneficial pattern as FOG [4–7].

## Conclusions

Overall, LFS of 60 Hz seems to consistently work well in patients with FOG at HFS of bilateral STN DBS to improve FOG, speech and swallowing function and other axial symptoms, though tremor could worsen compared with HFS in a minority of patients. We are still not very certain about the exact effect of LFS and its underlying mechanism of benefit due to the small participant size and small number of studies on LFS versus HFS, with only a few randomized, double blind, prospective studies, which is further complicated by other variables differing across studies as discussed. Whether LFS simply addresses the frequency specific axial symptoms in the context of HFS or has other beneficial effect requires more studies, such as comparing LFS to DBS Off states in more patients with FOG at HFS or characterizing  the locations of the DBS electrodes and active contacts ﻿in the context of PPNa or its pathway ﻿in patients with or without FOG, which might also possibly help understand the underlying mechanism in the future.

## Abbreviations

FOG: Freezing of gait
HFS: High frequency stimulation
LFS: Low frequency stimulation
PD: Parkinson disease
PPNa: Pedunculopontine nucleus area
STN DBS: Subthalamic nucleus deep brain stimulation
SWS: Stand-Walk-Sit test
TEED: Total electric energy delivered
UPDRS-III: Unified Parkinson disease rating scale section III for motor function
